# Assessment of Illness Severity in Adults Hospitalized With Acute Respiratory Tract Infection due to Influenza, Respiratory Syncytial Virus, or Human Metapneumovirus

**DOI:** 10.1111/irv.13275

**Published:** 2024-05-01

**Authors:** Ann R. Falsey, Edward E. Walsh, Stacey L. House, Yannick Vandendijck, Marita Stevens, Eric K. H. Chan, Gabriela Ispas

**Affiliations:** ^1^ Department of Medicine University of Rochester Rochester New York USA; ^2^ Department of Emergency Medicine, School of Medicine Washington University St. Louis Missouri USA; ^3^ Janssen Research & Development Beerse Belgium; ^4^ Janssen Global Services, LLC Raritan New Jersey USA; ^5^ Janssen Global Medical Affairs Infectious Diseases & Vaccines Beerse Belgium

**Keywords:** HARTI, hMPV, hospitalization, human metapneumovirus, influenza, patient‐reported outcomes, respiratory syncytial virus, RSV

## Abstract

**Background:**

Influenza, respiratory syncytial virus (RSV), and human metapneumovirus (hMPV) are common respiratory viruses causing similar symptoms. Optimal tools to assess illness severity for these viruses have not been defined. Using the Hospitalized Acute Respiratory Tract Infection (HARTI) study data, we report symptom severity by clinician‐rated clinical severity scores (CSS) in adults with influenza, RSV, or hMPV and correlations between CSS and patient‐reported outcomes (PROs).

**Methods:**

HARTI was a global epidemiologic study in adults hospitalized with acute respiratory tract infections. Patients were assessed at enrollment within 24 h of admission with CSS and twice during hospitalization with CSS, Respiratory Infection Intensity and Impact Questionnaire™ (RiiQ™), and EQ‐5D‐5L. Data were summarized descriptively, stratified by pathogen and baseline and hospitalization characteristics. Domain (general, upper respiratory, and lower respiratory) and sign/symptom subscores are presented for CSS; sign/symptom subscores are presented for RiiQ™ results.

**Results:**

Data from 635 patients with influenza, 248 with RSV, and 107 with hMPV were included. At enrollment, total CSS and general and lower respiratory signs/symptoms (LRS) scores were higher for RSV and hMPV than influenza. Between‐pathogen differences were greatest for LRS scores. Dyspnea, rales/rhonchi, wheezing, and shortness of breath scores trended higher for RSV and hMPV than influenza. RiiQ™ scores for cough, fatigue, and short of breath were strongly correlated with corresponding clinician‐rated symptoms.

**Conclusions:**

These findings support the use of PROs (e.g., the RiiQ™) correlating with clinician assessments to gauge patient well‐being and aid patient management by accurately assessing respiratory illness severity due to RSV, hMPV, or influenza.

**Abbreviations**
ARTI: acute respiratory tract infection
BMI: body mass index
CI: confidence interval
COPD: chronic obstructive pulmonary disease
CRF: core risk factor
CSS: clinical severity scores
EQ‐5D‐5L: EuroQoL 5‐Dimension 5‐Level Health Assessment
HARTI: Hospitalized Acute Respiratory Tract Infection
hMPV: human metapneumovirus
HRQoL: health‐related quality of life
ICU: intensive care unit
LOS: length of stay
LRS: lower respiratory signs/symptoms
LRTI: lower respiratory tract infection
NEWS: National Early Warning Score
O_2_
: oxygen
PCR: polymerase chain reaction
PRO: patient‐reported outcome
RiiQ™: Respiratory Infection Intensity and Impact Questionnaire™
RSV: respiratory syncytial virus
SOC: standard of care
URS: upper respiratory signs/symptoms
VAS: visual analog scale

## 1 Introduction

Lower respiratory tract infections (LRTIs) are a leading contributor to mortality worldwide, causing an estimated 2.38 million deaths globally in 2016 [1]. Although SARS‐CoV‐2 has dramatically changed the landscape of LRTI, influenza and respiratory syncytial virus (RSV) remain important causes of serious adult illness. A systematic analysis of the global burden of disease in adults found that approximately 500,000 and 250,000 annual deaths were caused by influenza and RSV, respectively [2].

Human metapneumovirus (hMPV) is another important cause of acute respiratory tract infections (ARTIs) in adults [3]. A 4‐year prospective study found that, among those hospitalized with hMPV, 13% required intensive care unit (ICU) admission, 12% required ventilatory support, and 7% died during or shortly after hospitalization [4]. Like influenza and RSV, hMPV infections may cause acute exacerbations of asthma and chronic obstructive pulmonary disease (COPD) [5, 6].

Clinical presentation is similar for influenza, RSV, and hMPV infections, including in those at greatest risk for progression to severe disease (older adults [≥65 years], immunocompromised individuals, and those with underlying comorbidities, including asthma, COPD, cardiovascular disease, and diabetes) [7, 8]. The best tools to assess respiratory illness severity in adults have not been defined. Traditionally, specific outcomes, such as hypoxia, intensive care use, length of stay, or clinician assessments, have been utilized. Therefore, assessing correlations between clinician‐rated disease severity, patient‐reported outcomes (PROs) assessing symptom severity, and health‐related quality of life (HRQoL) could provide insight into which tools provide the most accurate measures of patient well‐being and the true burden of respiratory illness.

The Hospitalized Acute Respiratory Tract Infection (HARTI) study, conducted during the 2017–2019 epidemic seasons, assessed risk factors for severe disease and the distribution of respiratory pathogens in adults hospitalized with ARTIs [7]. Using data from the HARTI study, we report clinician‐rated ARTI disease severity throughout hospitalization and correlations between traditional clinician‐rated clinical severity scores (CSS) based on signs and symptoms, which were used as the reference standard, and PROs (Respiratory Infection Intensity and Impact Questionnaire™ [RiiQ™] and EuroQoL 5‐Dimension 5‐Level Health Assessment [EQ‐5D‐5L]) in adults hospitalized with influenza, RSV, or hMPV infections. Baseline CSS for those who were negative for influenza, RSV, and hMPV were also assessed.

## 2 Methods

### 2.1 Study Design

**FIGURE 1:**
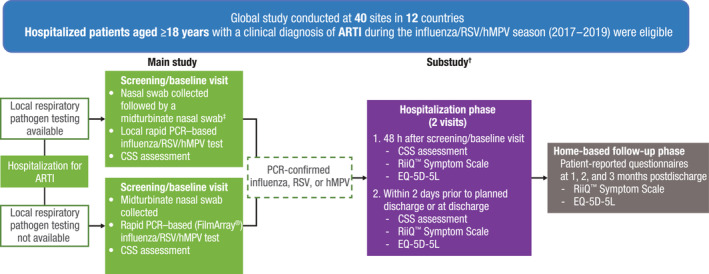
Study design. ARTI, acute respiratory tract infection; CSS, clinical severity scores; EQ‐5D‐5L, EuroQoL 5‐Dimension 5‐Level Health Assessment; PCR, polymerase chain reaction; RiiQ™, Respiratory Infection Intensity and Impact Questionnaire™; SOC, standard of care. ^†^CSS were recorded at the screening/baseline visit and two visits during the hospitalization phase; RiiQ™ Symptom Scale scores were recorded at two visits during the hospitalization phase and three visits during the home‐based follow‐up phase. Data for home‐based follow‐up are not shown here but have been previously reported [11]. ^‡^When the nasal swab was collected as part of SOC, an additional midturbinate swab was collected from the opposite nostril than the nostril used for an SOC test. Rapid PCR analysis was used to detect and identify respiratory pathogens from SOC nasal and midturbinate nasal swabs.

HARTI was a prospective global epidemiologic study conducted at 40 centers across 12 countries (Australia, Argentina, Brazil, Canada, France, Germany, Japan, Malaysia, Mexico, Republic of Korea, South Africa, and the United States) in adults (≥18 years) hospitalized with ARTIs [7]. Patients provided written informed consent and enrolled within 24 h after hospital admission (main study). Patients with polymerase chain reaction (PCR)–confirmed influenza, RSV, or hMPV were eligible for the substudy consisting of a hospitalization phase (maximum of three visits occurring at screening/baseline, 48 h after screening or at early discharge, and within approximately 2 days before planned hospital discharge). If a patient was hospitalized for <72 h or transferred to another ward prohibiting follow‐up, an early discharge assessment was performed, and assessments within 2 days of discharge were not available (Figure 1).

### 2.2 Data Collection

At screening, baseline demographic and clinical characteristics, including presence of core risk factors (CRFs) for progression to severe disease (i.e., those aged ≥65 years or with chronic heart disease, chronic renal disease, COPD, or asthma), and clinical severity based on National Early Warning Score (NEWS) were collected. NEWS were calculated using seven graded vital sign measurements (respiratory rate, oxygen saturation, oxygen supplementation, temperature, blood pressure, heart rate, and level of consciousness) as previously described [9]. Vital signs were each scored from 0 to 3, with total NEWS tabulated by summing vital sign scores (higher scores representing more severe disease [*low*: 0–4; *moderate*: 5–6 or an individual parameter score of 3; *high*: ≥7]). For all enrolled patients who provided informed consent, the level of consciousness was assumed to be “Alert” (i.e., score = 0).

Symptom severity was evaluated using clinician‐rated total CSS comprising a general symptoms domain (reflecting the bothersomeness of cough, sputum production, shortness of breath, and malaise), upper respiratory signs/symptoms (URS; nasal discharge, pharyngitis, sinus tenderness), and lower respiratory signs/symptoms (LRS; dyspnea, rales/rhonchi, wheezing) based on auscultation and clinical observation (Table S1). Each sign/symptom was rated on a scale of worsening severity from 0 to 3 points, with total CSS, general symptoms, URS, and LRS scores determined by adding individual scores (general symptoms, 12‐point maximum; URS and LRS, 9‐point maximum; CSS, 30‐point maximum). If an item was missing, the total and corresponding CSS domain scores were also missing. Assessments were conducted at screening/enrollment (main study) and during in‐hospital study visits (substudy). Patients with coinfections of influenza, RSV, and/or hMPV were excluded from the substudy analyses.

The RiiQ™ Symptom Scale and EQ‐5D‐5L were administered as interviews during in‐hospital study visits and follow‐up [10]. RiiQ™ Symptom Scale and EQ‐5D‐5L data from the HARTI study were previously reported [11]. Details pertaining to the RiiQ™ and EQ‐5D‐5L are provided in the supporting methods.

### 2.3 Statistical Analysis

Data collected from CSS assessments were summarized using descriptive statistics and frequency tabulations, stratified by pathogen, age group (18–39, 40–64, 65–74, and ≥75 years), presence of CRFs, length of hospital stay, need for oxygen supplementation during hospitalization, and ICU stay during hospitalization. For the main study, patients who were negative for influenza, RSV, and hMPV at screening were further subdivided into “no pathogen” and “other pathogen” (positive test for respiratory pathogens other than influenza, RSV, or hMPV) groups. Ninety‐five percent confidence intervals (CIs) were calculated for mean CSS; 95% CIs for proportions were calculated using Wilson's formula [12]. Kendall's tau‐b coefficient, a nonparametric correlation metric, was used to evaluate the strength of associations between individual CSS and RiiQ™ item scores [13]. Spearman correlation was used for association analysis between total CSS/subdomains and RiiQ™ domains or EQ‐5D‐5L. Means are reported in the text of the results, and 95% CIs are shown in the tables and figures.

## 3 Results

### 3.1 Main Study: Patients

Patient demographics for the main study were previously reported [7]. Overall, 3861 patients were included; the median (range) age was 63 (18–104) years, 53.6% were female, and 75.4% had CRFs. A total of 644 patients (16.7%) tested positive for influenza, 249 (6.4%) were positive for RSV, 107 (2.8%) had hMPV, and 6 (0.2%) presented with coinfections of influenza, RSV, and/or hMPV. At baseline, numerically higher proportions of patients with RSV (85.5%) and hMPV (85.0%) had CRFs than those with influenza (74.3%) or who tested negative for these three viruses (74.4%).

### 3.2 Main Study: CSS at Screening

**FIGURE 2:**
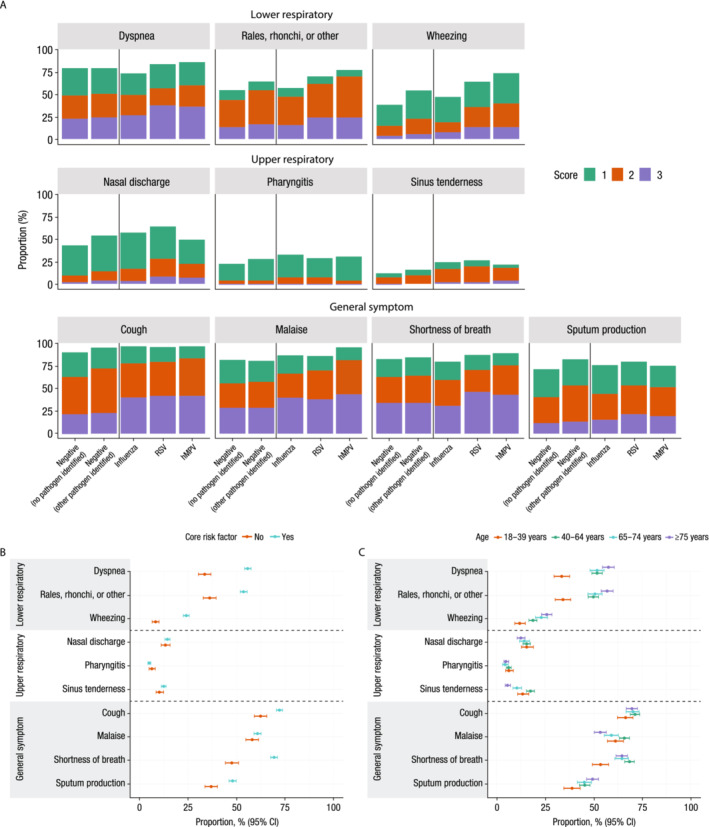
(A) Proportion of CSS individual items by pathogen^†^ with scores 1–3 and proportion of CSS individual items with scores of 2 or 3 by (B) presence of CRFs^‡^ or (C) age^§^ at screening (main study). CRF, core risk factor; CSS, clinical severity scores; hMPV, human metapneumovirus; RSV, respiratory syncytial virus. ^†^Represents the proportion of scores of the 10 CSS items (range of 0–3). Scores of 0 are not shown. Negative, no pathogen identified (*n* = 1762); negative, other pathogen identified (*n* = 684); influenza (*n* = 635); RSV (*n* = 248); hMPV (*n* = 107). ^‡^Represents the proportion of scores 2 and 3 for the 10 CSS items (range of 0–3). All negative, influenza, RSV, and hMPV group patients are pooled. Without CRF (*n* = 804); with CRF (*n* = 2632). ^§^Represents the proportion of scores 2 and 3 for the 10 CSS items (range of 0–3). All negative, influenza, RSV, and hMPV group patients are pooled. Age 18–39 years (*n* = 507); 40–64 years (*n* = 1265); 65–74 years (*n* = 686); ≥75 years (*n* = 978).

Complete CSS data were available for 635 patients (98.6%) with influenza, 248 (99.6%) with RSV, 107 (100%) with hMPV, and 2446 (99.8%) who tested negative (1762 with no other pathogen identified; 684 with another pathogen identified, the most frequent of which was enterovirus/rhinovirus [361 (52.8%)]; Table S2). The most commonly reported signs/symptoms at enrollment/screening included cough, malaise, dyspnea, shortness of breath, and sputum production, while the least commonly reported signs/symptoms were pharyngitis and sinus tenderness (Figure 2A). Numerically higher proportions of patients with RSV or hMPV had clinician‐reported rales/rhonchi and wheezing compared with those who had influenza or those with no pathogen identified. Additionally, numerically higher proportions of those with RSV or hMPV had scores of 2 or 3 for dyspnea, rales/rhonchi, wheezing, and shortness of breath compared with those who had influenza or no pathogen identified. Numerically higher proportions of patients with CRFs than without had individual sign/symptom scores of 2 or 3 for dyspnea, rales/rhonchi, wheezing, cough, shortness of breath, and sputum production (Figure 2B). Additionally, numerically higher proportions of patients aged ≥40 years had individual sign/symptom scores of 2 or 3 for dyspnea, rales/rhonchi, wheezing, shortness of breath, and sputum production compared with those aged 18–39 years (Figure 2C).

Mean total CSS were higher for patients with hMPV (14.5) or RSV (14.3) than those who had influenza (12.4) and those with no pathogen identified (10.8; Figure S1). Similar findings were observed for general symptom scores and LRS scores.

The highest mean total CSS were observed for patients who had CRFs, were female, were aged ≥40 years, were overweight or obese, or had moderate or high NEWS (Figure S2). Similar trends were observed for general symptom scores and LRS scores.

### 3.3 Substudy: Patient Characteristics

The substudy included 709 patients; among these, 366 (51.6%) had influenza, 238 (33.6%) had RSV, 100 (14.1%) had hMPV, and 5 (0.7%) had coinfections. The median (range) age of substudy patients was 67.0 (18–99) years, and 404 (57.0%) were female (Table 1). Overall, 570 patients (80.4%) had CRFs, including 276 (75.4%) with influenza, 205 (86.1%) with RSV, and 85 (85.0%) with hMPV.

**TABLE 1:** Baseline patient characteristics (substudy).

	Influenza (*n* = 366)	RSV(*n* = 238)	hMPV(*n* = 100)	Total(*N* = 709^a^)
Age, median (range), years	65.5 (18–99)	70.0 (18–98)	69.0 (24–93)	67.0 (18–99)
18–39, *n* (%)	29 (7.9)	18 (7.6)	7 (7.0)	55 (7.8)
40–64, *n* (%)	147 (40.2)	75 (31.5)	36 (36.0)	259 (36.5)
65–74, *n* (%)	90 (24.6)	61 (25.6)	22 (22.0)	174 (24.5)
≥75, *n* (%)	100 (27.3)	84 (35.3)	35 (35.0)	221 (31.2)
Gender, *n* (%)				
Female	193 (52.7)	147 (61.8)	62 (62.0)	404 (57.0)
Male	173 (47.3)	91 (38.2)	38 (38.0)	305 (43.0)
Presence of CRFs, *n* (%)	276 (75.4)	205 (86.1)	85 (85.0)	570 (80.4)
Age ≥65 years	190 (51.9)	145 (60.9)	57 (57.0)	395 (55.7)
Chronic heart disease	142 (38.8)	99 (41.6)	33 (33.0)	276 (38.9)
COPD	81 (22.1)	73 (30.7)	32 (32.0)	187 (26.4)
Chronic renal disease	46 (12.6)	39 (16.4)	22 (22.0)	109 (15.4)
Asthma	47 (12.8)	51 (21.4)	20 (20.0)	120 (16.9)

Abbreviations: COPD, chronic obstructive pulmonary disease; CRF, core risk factor; hMPV, human metapneumovirus; RSV, respiratory syncytial virus.

^a^ Includes five patients with coinfections (between influenza and RSV and hMPV).

### 3.4 Substudy: CSS During Hospitalization

Total CSS and subdomain scores were assessed for >98% of patients at screening/baseline, nearly 80% of patients at 48 h after screening, and approximately 37% of patients at 2 days predischarge (Table S3).

**FIGURE 3:**
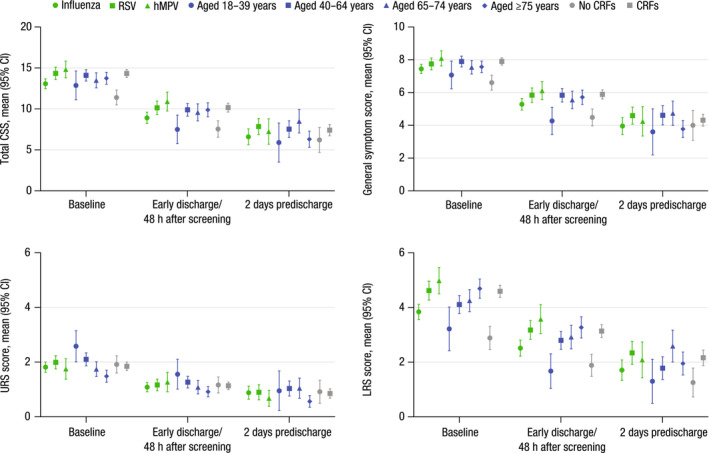
CSS over time by pathogen, age group, and presence of CRFs (substudy). CI, confidence interval; CRF, core risk factor; CSS, clinical severity scores; hMPV, human metapneumovirus; LRS, lower respiratory signs/symptoms; RSV, respiratory syncytial virus; URS, upper respiratory signs/symptoms.

Total CSS decreased linearly during hospitalization for all pathogens (Figure 3). Mean CSS for patients with influenza decreased from 13.0 at baseline to 6.6 (49.2%) at 2 days prior to discharge, while total CSS for those with RSV and hMPV decreased from 14.3 to 7.8 (45.5%) and 14.8 to 7.2 (51.4%), respectively. On average, LRS scores were higher than URS scores and exhibited the largest differences between pathogens at all time points. Mean LRS scores for patients with influenza decreased from 3.8 at baseline to 1.7 (55.3%) at 2 days before discharge; LRS scores for patients with RSV decreased from 4.6 to 2.3 (50.0%), while scores for those with hMPV decreased from 5.0 to 2.1 (58.0%) over the same period. Mean URS scores were similar between pathogens at all time points, ranging from 1.8 to 2.0 at baseline and from 0.7 to 0.9 at 2 days before discharge.

At all time points analyzed, mean LRS scores for patients aged 18–39 years were lower than LRS scores for those aged 65–74 or ≥75 years (Figure 3). At baseline and early discharge/48 h after screening, general symptom scores were also lower for patients aged 18–39 years compared with patients ≥40 years.

For patients with CRFs, mean general symptom scores, LRS scores, and total CSS were ≥1 point higher at baseline and early discharge/48 h after screening compared with those without CRFs (Figure 3). At 2 days predischarge, mean LRS scores were 2.2 and 1.3 for patients with CRFs or without CRFs, respectively.

Mean LRS scores were ≥0.5 points higher for all assessments for patients with a length of stay (LOS) > 3 days compared with those who had shorter (≤3 days) hospital stays (Figure S3). Mean general symptom scores, LRS scores, and total CSS were >1 point higher at all time points assessed for those who received oxygen supplementation versus those who did not. No substantial differences in CSS were observed between substudy patients admitted or not admitted to the ICU.

The individual symptom scores that exhibited the highest values at baseline were cough, malaise, and shortness of breath (Figure S4). For respiratory signs/symptoms, including dyspnea, rales/rhonchi, wheezing, and shortness of breath, there was a trend toward higher scores for patients with RSV or hMPV versus those who had influenza.

At baseline, patients with CRFs exhibited higher mean scores for dyspnea (1.9), rales/rhonchi (1.6), wheezing (1.2), cough (2.3), sputum production (1.5), and shortness of breath (2.0) than those without CRFs (dyspnea: 1.2; rales/rhonchi: 1.1; wheezing: 0.6; cough: 1.9; sputum production: 1.2; shortness of breath: 1.4; Figure S5). From screening to 2 days before planned discharge, scores for all clinical signs/symptoms decreased for all pathogen groups, all age groups, and in patients with or without CRFs.

### 3.5 Correlations Between Clinician‐Rated (CSS) and Patient‐Reported (RiiQ™ and EQ‐5D‐5L) Outcomes

**FIGURE 4:**
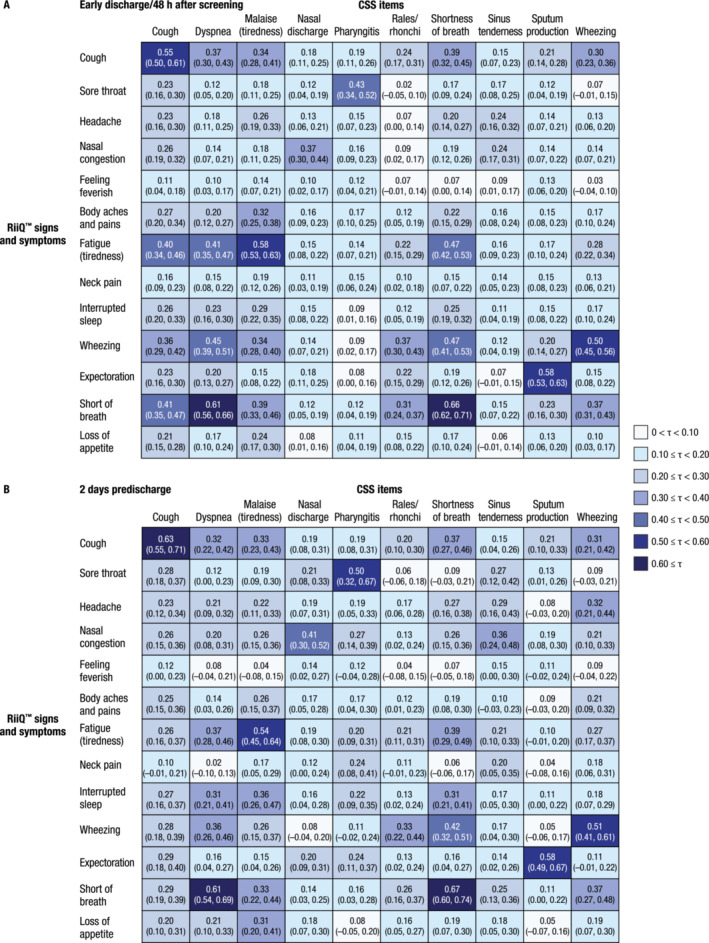
Kendall's tau correlation coefficient (95% CI) between individual CSS and RiiQ™ item scores in substudy patients evaluated at (A) early discharge/48 h after screening and (B) 2 days predischarge. CSS, clinical severity scores; RiiQ™, Respiratory Infection Intensity and Impact Questionnaire™.

RiiQ™ Symptom Scale and EQ‐5D‐5L data were collected at early discharge/48 h after screening and 2 days prior to discharge (Table S3). Several clinician‐rated individual symptom scores were strongly (τ ≥ 0.4) or very strongly (τ ≥ 0.6) correlated with those rated by patients through the RiiQ™ (Figure 4).

For example, clinician‐ and patient‐rated scores for cough were strongly correlated at early discharge/48 h after screening and 2 days prior to discharge. At early discharge/48 h after screening and at 2 days prior to discharge, CSS for dyspnea and shortness of breath were very strongly correlated with RiiQ™ scores for short of breath.

The clinician‐rated signs/symptoms of cough, dyspnea, malaise, and shortness of breath exhibited strong correlations with RiiQ™ scores for fatigue at early discharge/48 h after screening.

Additional strongly correlated CSS/RiiQ™ items at early discharge/48 h after screening included sputum production/expectoration, dyspnea/wheezing, shortness of breath/wheezing, and wheezing/wheezing. In general, these correlations remained consistent and strong (except for dyspnea/wheezing) at 2 days prior to discharge. Finally, strong correlations existed between pharyngitis/sore throat at early discharge/48 h after screening and 2 days prior to discharge.

**FIGURE 5:**
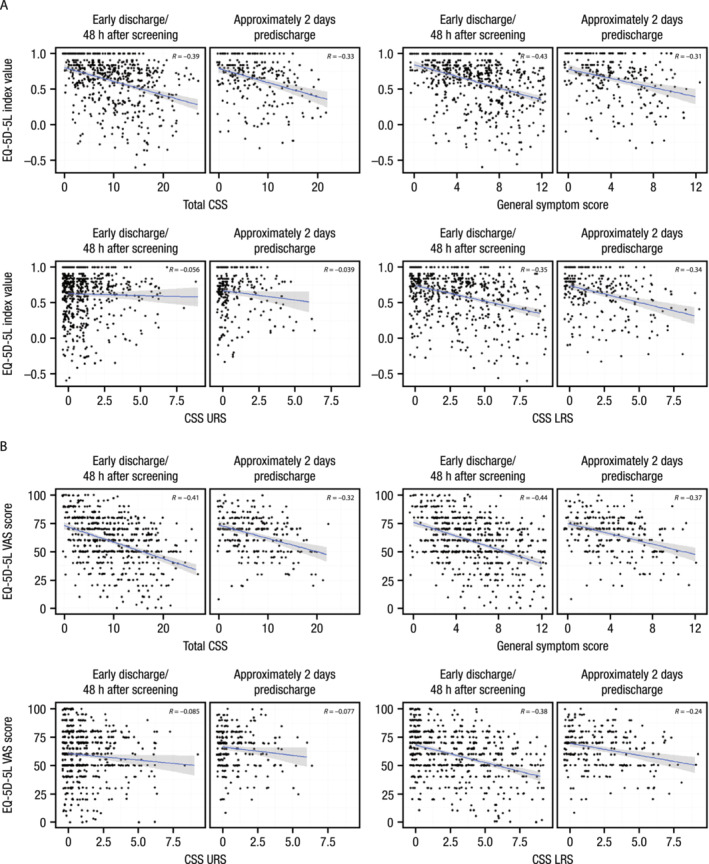
Scatterplot and Spearman correlations between CSS and EQ‐5D‐5L (A) index values and (B) VAS scores (substudy). CSS, clinical severity scores; EQ‐5D‐5L, EuroQoL 5‐Dimension 5‐Level Health Assessment; LRS, lower respiratory signs/symptoms; URS, upper respiratory signs/symptoms; VAS, visual analog scale.

During hospitalization, general symptom scores (*R* = −0.43 to −0.31), total CSS (*R* = −0.39 to −0.33), and LRS scores (−0.35 to −0.34) exhibited the strongest associations with EQ‐5D‐5L index values, while URS scores exhibited no association (−0.06 to −0.04; Figure 5A and Table S4). Similar trends were observed for EQ‐5D‐5L visual analog scale (VAS) scores (Figure 5B and Table S4).

## 4 Discussion

This subanalysis of HARTI study data aimed to describe the clinical manifestations of influenza, RSV, and hMPV in hospitalized adults using clinician‐ and patient‐reported sign and symptom severity scores. At screening, clinician‐rated total CSS, general symptom scores, and LRS scores were higher for those with RSV or hMPV compared with those who had influenza or no pathogen identified. During hospitalization, patients with RSV or hMPV remained more symptomatic as reflected by clinician‐ and patient‐reported disease severity than those with influenza, with LRS scores exhibiting the biggest differences in disease severity between pathogen groups throughout hospitalization. These findings are consistent with other analyses that reported RSV and hMPV hospitalizations require similar or greater medical resource utilization compared with influenza hospitalizations [7, 14]. Total CSS and LRS scores were also higher in older patients, patients with CRFs, patients with hospital stays >3 days, and patients who needed oxygen supplementation during hospitalization.

Patient management and categorization of severe ARTIs require considerable, high‐cost resources, including chest imaging, oxygen supplementation, and blood work, some of which may not be accessible at the earliest stages of patient care [15]. Furthermore, with the expansion of telemedicine and opportunities for remote patient monitoring, direct‐to‐patient solutions could be implemented in the future. In this context, understanding how patient‐reported signs/symptoms correlate with traditional clinician‐reported signs/symptoms and resource utilization is of utmost importance. To our knowledge, this is the first concurrent evaluation of both clinician‐ and patient‐reported signs/symptoms of disease in adults hospitalized with ARTIs.

Considering disease severity as reported by clinicians (CSS) as the gold standard, patient‐reported disease severity (RiiQ™) showed strong and very strong correlations, not only for defined intracomponents (cough, malaise/fatigue, wheezing, sputum production/expectoration, and shortness of breath) but also for paired intercomponents (pharyngitis/sore throat, shortness of breath/fatigue, cough/fatigue, dyspnea/fatigue, dyspnea/wheezing, shortness of breath/wheezing, cough/shortness of breath, and dyspnea/shortness of breath). Furthermore, our findings suggest that the severity of core signs/symptoms (i.e., cough, sputum production/expectoration, shortness of breath/dyspnea, fatigue, and wheezing) was similarly reported by clinicians and patients and that higher severity was associated with increased requirement for oxygen supplementation. Overall, these findings warrant further research for the development of signs/symptoms‐based algorithms to support management of adult ARTIs, potentially through telemedicine.

A previous analysis of HARTI substudy patients reported that RiiQ™ Symptom Scale scores were moderately associated with EQ‐5D‐5L index value scores [11]. In the current analysis, CSS and EQ‐5D‐5L (both index values and VAS scores) were negatively associated at both early discharge/48 h after screening and 2 days before discharge. The negative association between CSS and EQ‐5D‐5L is expected given that increased symptom severity is likely to negatively impact patients' self‐rated health. The finding that CSS and EQ‐5D‐5L were negatively associated for general symptom and LRS scores but not URS scores suggests that general and lower respiratory symptoms may be the primary drivers of reduced HRQoL during ARTI.

A major strength of this study was the large number of patients, allowing for assessments of disease severity through clinician‐rated symptoms and multiple PROs. Furthermore, this study was conducted in adults ≥18 years of age, allowing for comparisons between younger and older adult populations. Study limitations include the wide variation in the positivity rate for the respiratory pathogens of interest across sites. For example, the positivity rate for pathogens of interest for sites in the United States ranged from 22.6% to 93.4%. This indicates that prescreening likely occurred for sites utilizing their standard of care (SOC) viral detection test. Finally, detection of other respiratory pathogens causing ARTIs using multiplex PCR assay was not standardized and was conducted either centrally or locally depending on the study site's available equipment. Sensitivity of the PCRs used by study sites may have differed from the central multiplex panel. In addition, there may have been discrepancies in respiratory panels for patients with tests conducted via SOC versus patients with tests conducted using kits provided by the study sponsor. Thus, the study was not designed to assess relative burden as a cause of hospitalization of the respiratory pathogens of interest.

In conclusion, these results suggest that, among adults hospitalized with respiratory viral pathogens, symptom severity for RSV and hMPV may be greater than for influenza, particularly for older adults and those with CRFs. The correlation of traditional assessments of disease severity by clinicians with PRO tools, such as the RiiQ™, supports their use to provide comprehensive evaluations of patient well‐being for accurate assessment of respiratory illness severity. Such tools may prove beneficial in the management of adult ARTIs, potentially including triage, in research and clinical settings.

## Author Contributions

**Ann R. Falsey:** Investigation; Writing – review and editing. **Edward E. Walsh:** Investigation; Writing – review and editing. **Stacey L. House:** Investigation; Writing – review and editing. **Yannick Vandendijck:** Conceptualization; Formal analysis; Methodology; Software; Visualization; Writing – original draft; Writing – review and editing. **Marita Stevens:** Conceptualization; Methodology; Writing – original draft; Writing – review and editing. **Eric K. H. Chan:** Formal analysis; Writing – review and editing. **Gabriela Ispas:** Conceptualization; Formal analysis; Funding acquisition; Methodology; Supervision; Writing – original draft; Writing – review and editing.

## Ethics Statement

All studies were approved by the human investigational review board or ethics committee of each study center. All studies were carried out in accordance with the principles of the Declaration of Helsinki.

## Consent

Patients gave written informed consent prior to enrollment in the study.

## Conflicts of Interest

**ARF** has received research grants from Janssen, Merck Sharp & Dohme, Pfizer, BioFire Diagnostics, Moderna, Vax Co., and CyanVac; consulting fees from Sanofi Pasteur, ADMA Biologics, and Arrowhead; and personal fees for serving on a Data Safety Monitoring Board for Novavax. **EEW** received grants from Merck, Janssen, and Pfizer and was a paid member of the Data Safety and Monitoring Board for GSK. **SLH** received advisory board consulting fees from Janssen Pharmaceuticals. **YV** is an employee of Janssen Research & Development. **MS** is a shareholder of Johnson & Johnson and former employee of Janssen Research & Development. **EKHC** is an employee of Janssen Global Services, LLC, and is a shareholder of Johnson & Johnson. **GI** is a shareholder of Johnson & Johnson and a former employee of Janssen Global Medical Affairs Infectious Diseases & Vaccines.

## Supporting information


**Table S1.** Clinical severity scores: signs and symptoms by domain.
**Table S2.** Patients with complete CSS at screening (main study).
**Table S3.** Patients with CSS, RiiQ™, and EQ‐5D‐5L data available (substudy).
**Table S4.** Spearman correlations (95% CI) between CSS and EQ‐5D‐5L scores at early discharge/48 h after screening and 2 days predischarge (substudy).

## Data Availability

Although these data are not currently publicly available for sharing, requests for sharing can be sent to the corresponding author and will be evaluated on an individual basis.
