# Supplementary material for: Assessment of Illness Severity in Adults Hospitalized With Acute Respiratory Tract Infection due to Influenza, Respiratory Syncytial Virus, or Human Metapneumovirus
Source: Influenza Other Respir Viruses. 2024 May 1;18(5):e13275. doi: 10.1111/irv.13275 (PMC11062776; doi:10.1111/irv.13275)
Supplement: Supplementary file 1 — Table S1. Clinical severity scores: signs and symptoms by domain. Table S2. Patients with complete CSS at screening (main study). Table S3. Patients with CSS, RiiQ™, and EQ‐5D‐5L data available (substudy). Table S4. Spearman correlations (95% CI) between CSS and EQ‐5D‐5L scores at early discharge/48 h after screening and 2 days predischarge (substudy). [file IRV-18-e13275-s001.docx]

**Supporting Information**

**Assessment of Illness Severity in Adults Hospitalized With Acute Respiratory Tract Infection Due to Influenza, Respiratory Syncytial Virus, or Human Metapneumovirus**

Ann R. Falsey,^1^ Edward E. Walsh,^1^ Stacey L. House,^2^ Yannick Vandendijck,^3^ Marita Stevens,^3^
Eric K. H. Chan,^4^ Gabriela Ispas^5^

^1^University of Rochester School of Medicine, Rochester, New York, USA; ^2^Department of Emergency Medicine, Washington University School of Medicine, St. Louis, MO, USA; ^3^Janssen Research & Development, Beerse, Belgium; ^4^Janssen Global Services, LLC, Raritan, NJ, USA; ^5^Janssen Global Medical Affairs Infectious Diseases & Vaccines, Beerse, Belgium

**Supporting Methods**

*Data Collection*

The Respiratory Infection Intensity and Impact Questionnaire™ (RiiQ™) is a patient-reported outcome (PRO) tool used in this study for assessing lower respiratory (cough, wheezing, expectoration, and short of breath), upper respiratory (sore throat and nasal congestion), and systemic (feeling feverish, headache, neck pain, fatigue, loss of appetite, interrupted sleep, and body aches and pains) symptoms at their worst during the past 24 hours on a scale from 0 (none) to 3 (severe). Domain scores were calculated by averaging symptom scores across each domain. Total RiiQ™ score was calculating by summing the scores of all 13 items.

The EuroQoL 5-Dimension 5-Level Health Assessment (EQ-5D-5L) measures health-related quality of life (HRQoL) based on 5 dimensions (mobility, self-care, usual activities, pain/discomfort, and anxiety/depression), rated on 5 levels (ranging from “No Problems” to “Extreme Problems”).^1^ The EQ-5D visual analog scale (EQ-VAS) records patients’ overall self-rated health, with values between 100 (best imaginable health) and 0 (worst imaginable health). EQ-5D-5L health states, defined by the EQ-5D-5L descriptive system, were converted into a single index value using the time trade-off method and United Kingdom value set.^2,3^ If a patient could not be interviewed (eg, was too ill to complete the questionnaire), RiiQ™ Symptom Scale and EQ-5D-5L were not collected.

**Table S1.** Clinical severity scores: signs and symptoms by domain.

|  | | **Score** | | | |
| --- | --- | --- | --- | --- | --- |
|  |  | **0** | **1** | **2** | **3** |
| **General symptom score** | Cough, sputum production, shortness of breath, malaise | No symptoms | Just noticeable | Bothersome sometimes, not interfering with other activities | Bothersome most of the time, interfering with other activities |
| **Upper respiratory score** | Nasal discharge | None | Clear, serous, scant, but slightly increased | Clear to white, obvious increased volume, and minor blood streaks on tissue | Frankly purulent (yellow or green) or gross blood |
|  | Pharyngitis | None | Mild and/or patchy erythema | Marked and/or confluent erythema | Erythema and purulent exudate |
|  | Sinus tenderness | None | Not applicable | Mild tenderness | Severe tenderness or overlying erythema |
| **Lower respiratory score** | Dyspnea | None | May have brief episodes (minutes to max 1 hour) | May have increased episodes (longer than 1 hour but less than 1 day) | May have long episodes (lasting longer than 1 day) |
|  | Rales, rhonchi, or other | None | Not applicable | Scattered wheezes or rhonchi | Widespread wheezes or rhonchi, rales, dyspnea, or signs of consolidation |
|  | Wheezing | None | Terminal expiration or only with stethoscope | Entire expiration or audible on expiration without stethoscope | Inspiration and expiration without stethoscope |

**Table S2.** Patients with complete CSS at screening (main study).

|  | **Influenza**  **(n = 644)** | **RSV**  **(n = 249)** | **hMPV**  **(n = 107)** | **Negative test**  **(n = 2450)^†^** | **Coinfection**  **(n = 6)** | **Total^‡^**  **(n = 3456)** |
| --- | --- | --- | --- | --- | --- | --- |
| Complete CSS available, n (%) | 635 (98.6) | 248 (99.6) | 107 (100.0) | 2446 (99.8)^§^ | 6 (100.0) | 3442 (99.6) |

CSS, clinical severity scores.

^†^Among patients with negative tests, 362 (14.8%) had enterovirus/rhinovirus, 124 (5.1%) had coronaviruses, 103 (4.2%) had streptococcus pneumonia, 83 (3.4%) had parainfluenza, and 68 (2.8%) had other pathogens.

^‡^Patients without data on other pathogens, with negative tests but with herpes simplex only, or who had influenza, RSV, or hMPV on the off-site respiratory panel were excluded (n = 405). Includes negative test patients without other pathogens identified.

^§^Includes those with information on other pathogens identified (n = 684), including coronavirus, enterovirus/rhinovirus, parainfluenza viruses, and no other pathogen identified (n = 1762).

**Table S3.** Patients with CSS, RiiQ™, and EQ-5D-5L data available (substudy).

|  | **Baseline** | **Early**  **discharge** | **48h after screening** | **2 days predischarge** | |
| --- | --- | --- | --- | --- | --- |
| **CSS, n (%)** | **N=709** | | | | |
| Total CSS | 701 (98.9) | 54 (7.6) | 550 (77.6) | | 262 (37.0) |
| General symptoms | 706 (99.6) | 54 (7.6) | 558 (78.7) | | 266 (37.5) |
| URS | 702 (99.0) | 61 (8.6) | 561 (79.1) | | 274 (38.6) |
| LRS | 706 (99.6) | 58 (8.2) | 563 (79.4) | | 276 (38.9) |
| **RiiQ™, n (%)** |  | 67 (9.4) | 536 (75.6) | | 252 (35.5) |
| **ED-5D-5L index values, n (%)** |  | 66 (9.3) | 536 (75.6) | | 252 (35.5) |
| **EQ-5D-5L VAS, n (%)** |  | 66 (9.3) | 531 (74.9) | | 252 (35.5) |

CSS, clinical severity scores; EQ-5D-5L, 5-level EQ-5D; LRS, lower respiratory signs/symptoms; RiiQ™, Respiratory Infection Intensity and Impact Questionnaire™; URS, upper respiratory signs/symptoms; VAS, visual analogue scale.

**Table S4.** Spearman correlations (95% CI) between CSS and EQ-5D-5L scores at early discharge/48h after screening and 2 days predischarge (substudy).

|  |  | **Early discharge/48 h after screening** | | **2 days predischarge** | |
| --- | --- | --- | --- | --- | --- |
|  |  | **EQ-5D-5L** | | **EQ-5D-5L** | |
|  |  | **Index value** | **VAS score** | **Index value** | **VAS score** |
| **CSS** | **Total** | –0.39 (–0.45, –0.31) | –0.41 (–0.48, –0.34) | –0.33 (–0.43, –0.21) | –0.32 (–0.43, –0.21) |
|  | **LRS** | –0.35 (–0.42, –0.28) | –0.38 (–0.45, –0.31) | –0.34 (–0.45, –0.23) | –0.24 (–0.35, –0.11) |
|  | **URS** | –0.06 (–0.14, 0.03) | –0.08 (–0.17, 0.00) | –0.04 (–0.16, 0.09) | –0.08 (–0.20, 0.05) |
|  | **General symptoms** | –0.43 (–0.49, –0.36) | –0.44 (–0.50, –0.37) | –0.31 (–0.41, –0.19) | –0.37 (–0.47, –0.26) |

CI, confidence interval; CSS, clinical severity scores; EQ-5D-5L, 5-level EQ-5D; LRS, lower respiratory signs/symptoms; URS, upper respiratory signs/symptoms; VAS, visual analog scale.

**Figure S1.** Clinical severity scores domain scores for total CSS, general symptom score, URS score, and LRS score by pathogen at screening (main study).


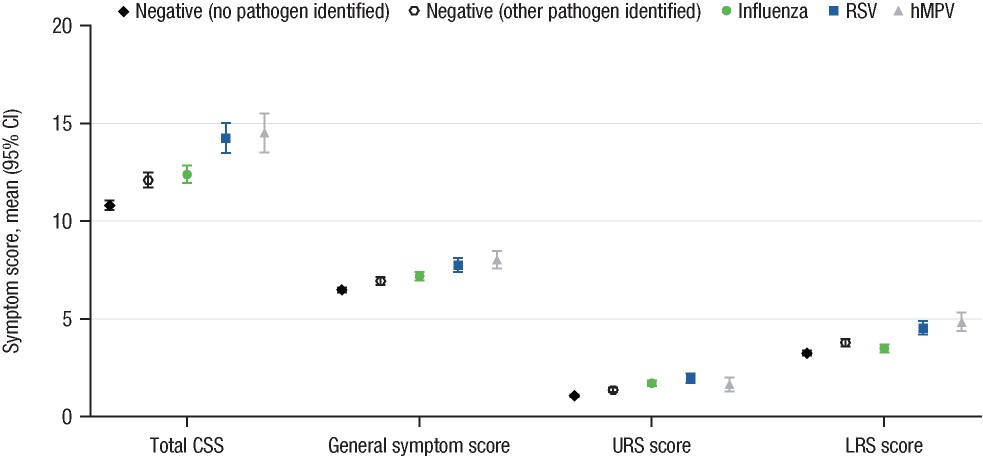


CI, confidence interval; CSS, clinical severity scores; hMPV, human metapneumovirus; LRS, lower respiratory signs/symptoms; RSV, respiratory syncytial virus; URS, upper respiratory signs/symptoms.

**Figure S2.** CSS domains at screening for (A) total CSS, (B) general symptom score, (C) URS score, and (D) LRS score by subgroup (main study).


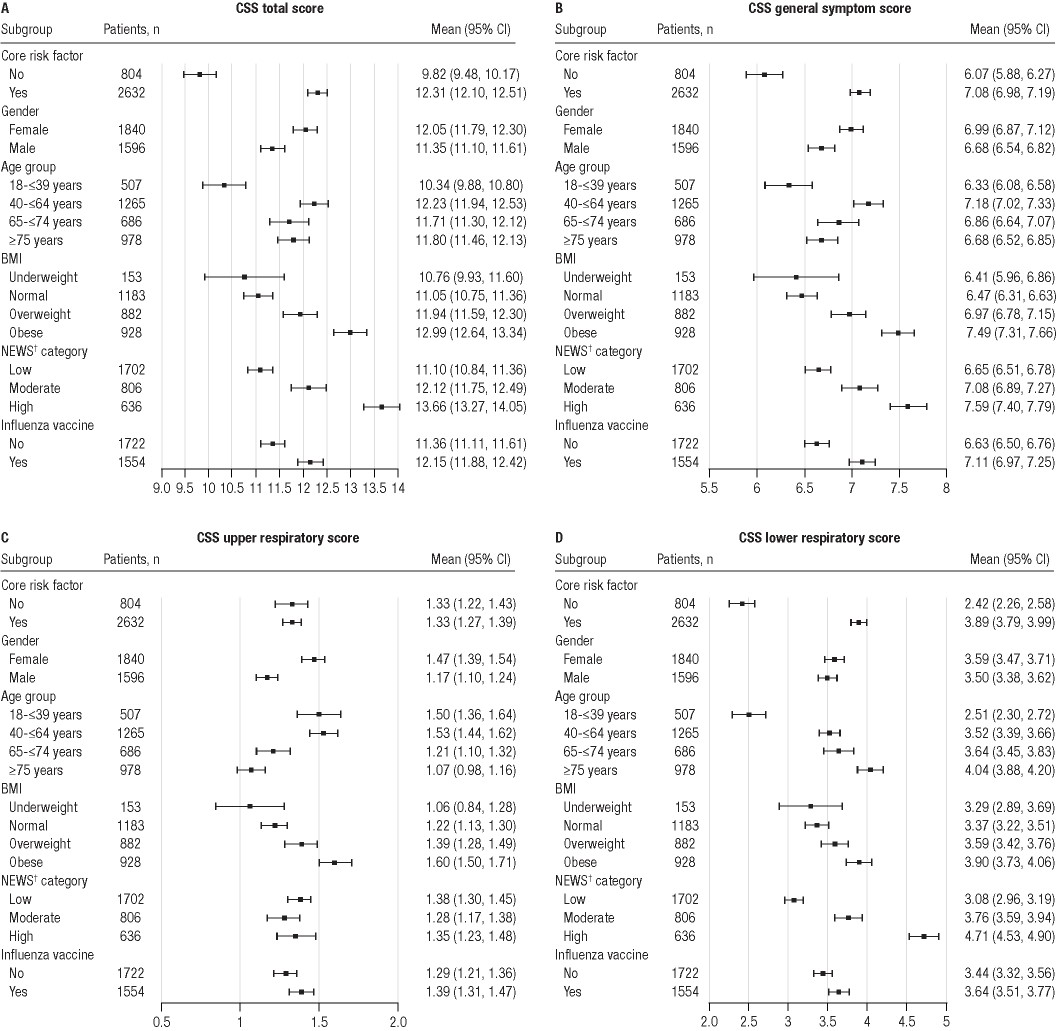


BMI, body mass index; CRF, core risk factor; CSS, clinical severity scores; LRS, lower respiratory signs/symptoms; NEWS, National Early Warning Score; URS, upper respiratory signs/symptoms.

^†^At screening, NEWS were calculated using 7 graded vital sign measurements (respiratory rate, oxygen saturation, oxygen supplementation, temperature, blood pressure, heart rate, and level of consciousness) as previously described.^4^ Each vital sign was scored from 0 to 3; total NEWS were calculated by summing vital sign scores, with higher scores representing more severe disease (low: 0 to 4; moderate: 5 to 6 or an individual parameter score of 3; high: ≥7). For all enrolled patients who provided informed consent, the level of consciousness was assumed to be “Alert” (ie, score = 0).

**Figure S3.** CSS (mean [95% CI]) over time by LOS, oxygen supplementation, and admission to ICU (substudy).


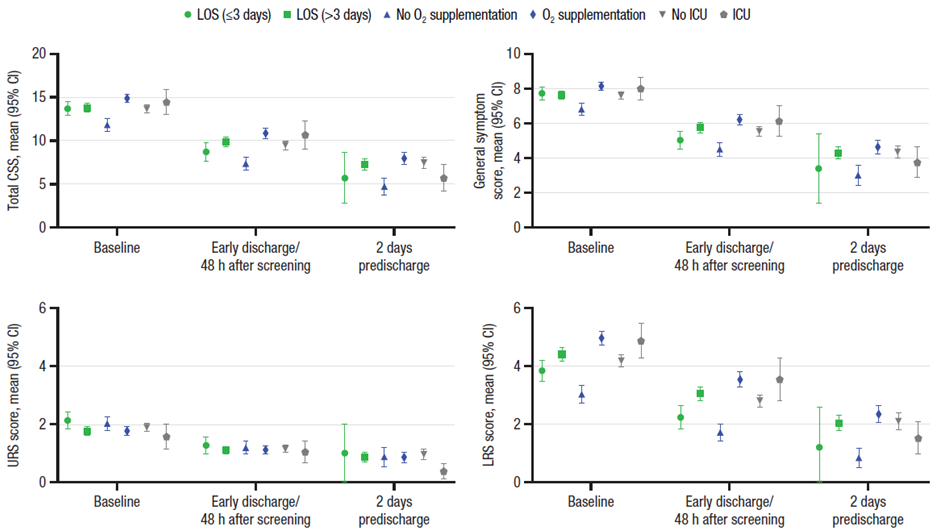


CI, confidence interval; CSS, clinical severity scores; ICU, intensive care unit; LOS, length of stay; LRS, lower respiratory signs/symptoms; O_2_, oxygen; URS, upper respiratory signs/symptoms.

**Figure S4.** Individual symptom scores (mean [95% CI]) over time by pathogen (substudy).


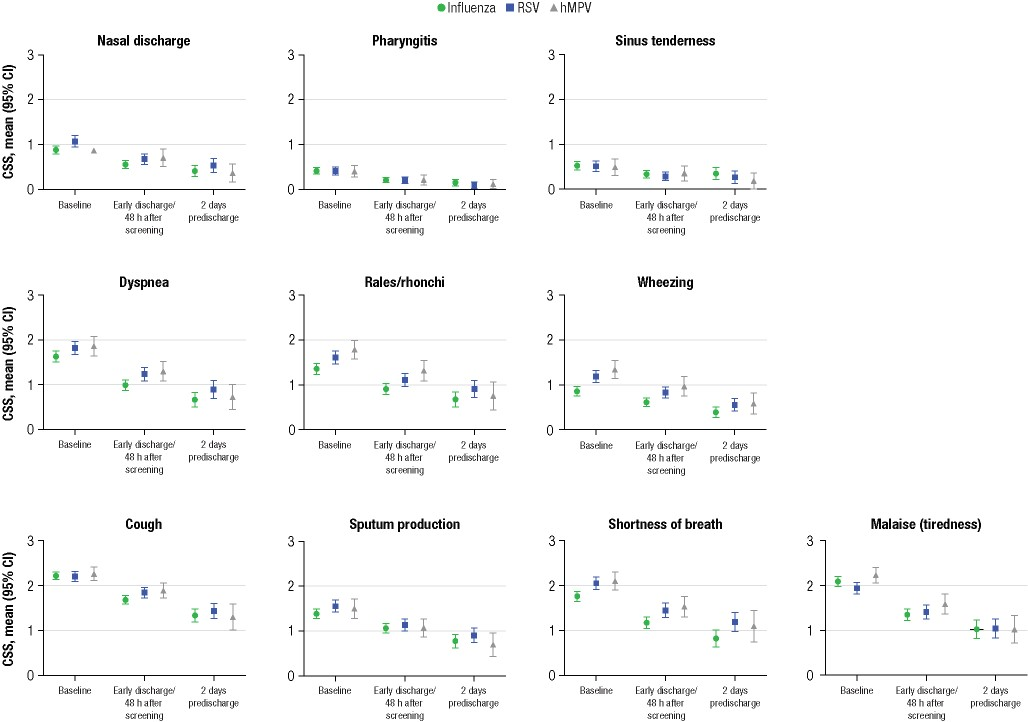


CI, confidence interval; CSS, clinical severity scores; hMPV, human metapneumovirus; RSV, respiratory syncytial virus.

**Figure S5.** Individual symptom scores (mean [95% CI]) over time by age and CRFs (substudy).


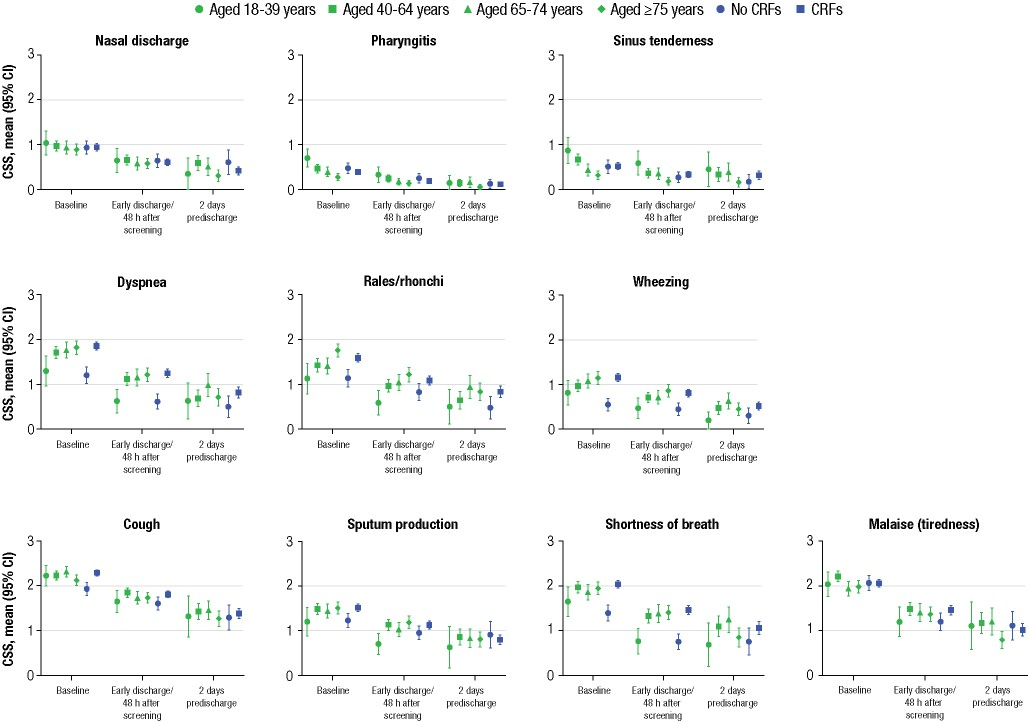


CI, confidence interval; CRF, core risk factor; CSS, clinical severity scores.

**Supplementary References**

1. EuroQol. About EQ-5D-5L. Accessed March 7, 2022. https://euroqol.org/eq-5d-instruments/eq-5d-5l-about/
2. Oemar M, Janssen B. EQ-5D-5L user guide: how to apply, score, and present results from the EQ-5D-Y. Version 2.0. Accessed October 2, 2023. https://euroqol.org/publications/user-guides/
3. van Hout B, Janssen MF, Feng YS, et al. Interim scoring for the EQ-5D-5L: mapping the EQ-5D-5L to EQ-5D-3L value sets. *Value Health* 2012;15(5):708-715.
4. National Health Service. National Early Warning Score (NEWS). Accessed December 5, 2022. <https://www.england.nhs.uk/ourwork/clinical-policy/sepsis/nationalearlywarningscore/>
